# SOAR Online Course Increases Capacity for Assisting Individuals with Disabilities in the US

**DOI:** 10.3389/fpubh.2016.00104

**Published:** 2016-05-26

**Authors:** Kristin Lupfer, Jen Elder

**Affiliations:** ^1^Substance Abuse and Mental Health Services Administration (SAMHSA) SOAR TA Center, Policy Research Associates Inc., Delmar, NY, USA

**Keywords:** homelessness, disability, social security, SSI, SSDI, mental illness, substance use disorders

## Abstract

For adults with disabilities who are experiencing homelessness, chances of being approved for social security disability benefits are very low, without assistance. Assisting with the Supplemental Security Income (SSI)/Social Security Disability Insurance (SSDI) application process can be challenging for case managers who lack capacity and expertise. Training caseworkers to document disability and submit complete, high-quality applications using the SSI/SSDI Outreach, Access and Recovery (SOAR) model improves efficiency and outcomes. Nationally, 65% of applications using the SOAR model are approved, with decisions received in an average of 81 days in 2015. The SOAR Online Course was created to expand training opportunities for individuals to learn how to effectively assist with SSI/SSDI applications for individuals experiencing or at risk for homelessness. From October 1, 2014 to September 30, 2015, 1049 individuals from 49 states, Washington, DC, and Puerto Rico successfully completed the SOAR Online Course. The course is a unique public health training model; in that, it incorporates a realistic and multimodal practice SSI/SSDI application with comprehensive feedback provided by experts. Local SOAR leaders around the county are trained to facilitate and guide groups through the course. This study evaluated data on online course usage, user experience, and the translation from learning to practice for online course trainees. We found that successful course completions were most concentrated in areas that had local SOAR leaders, trainees through the online course had higher data entry rates about case outcomes in the SOAR Online Application Tracking system, and that trainees reported a high satisfaction rate with the course and comprehensive feedback. The evaluation found that key success factors for online training models include the integration of a practice case component (or other generative learning activity), support from local facilitators, and feedback and technical assistance for trainees.

## Introduction

SSI/SSDI Outreach, Access and Recovery (SOAR) is an approach that helps states increase access to mainstream benefits for people who are experiencing or at risk of homelessness through strategic planning, training, and technical assistance.

The SOAR TA initiative evolved to address barriers to the receipt of Supplemental Security Income (SSI) and Social Security Disability Insurance (SSDI) among adults with disabilities who were experiencing homelessness. The correlation between disabilities and homelessness is high. Forty-two percent of individuals experiencing homelessness in 2013 reported having a severe mental illness or chronic substance use problem (1). For persons with mental illnesses who are homeless, SSA’s disability programs can provide a steady source of income (2) and health insurance, making it possible for many to secure housing, treatment, and other needed supports (3, 4). Research into the social determinants of mental health has shown that improving housing can improve depression and stress (5). Additionally, individuals who have stable housing are more likely to remain in treatment and successfully complete substance use recovery programs (6).

Despite high levels of disability among people who are homeless, many who are potentially eligible never apply for benefits (7). Among those who apply, the chance of being approved for SSI/SSDI is very low without someone taking an active role to assist with documentation of disability. Nationwide, initial approvals of SSI/SSDI applications, whether the applicant is homeless or not, average 28% (8). Denials typically result from SSA’s inability to contact the individual, missed appointments, and, more generally, the lack of adequate documentation. Once an application is denied, the wait for a hearing at the administrative law judge (ALJ) level, where two-thirds of appellants are approved, averages 15 months (9).

People experiencing homelessness face many challenges when applying to SSA for disability benefits (10). These challenges generally fall into two types: those related to physical and mental health problems and the nature of homelessness and those related to more systemic issues. People who are homeless are more likely than those who have never been homeless to have serious mental illnesses alone or in combination with other disabilities, such as cognitive disorders, chronic physical health conditions, and substance use disorders (11). Disability based on a mental illness or cognitive disorder is often more difficult to document than other disabilities due to inconsistent treatment histories and difficulty in finding medical records for people who may have been treated in many places over a long time. Beyond these individual-level challenges, barriers inherent in the system of care also exacerbate a person’s difficulty in applying for benefits. Chief among these is that most case managers are unable to assist people who are homeless with SSI/SSDI because they have neither the time nor the expertise. Case managers who try to help applicants are frustrated by a lack of understanding about how to make the process work more effectively.

Furthermore, the SSI/SSDI application process is time-intensive, time-sensitive, and very complex. Case managers have only 60 days from the protective filing date to navigate the complex web of requirements and complete all necessary components of the SSA packet on behalf of their clients. Case managers must then track multiple requests for information and submission of necessary information, while frequently meeting clients out in the community and often working in multiple locations in a day. For these reasons, proper training and support are critical to ensure approval. By helping increase caseworker productivity and, in turn, improving approval rates and decreasing processing times on SSI/SSDI applications, the SOAR Online Course has far-reaching benefits, which specifically address the challenges we face with an insufficient workforce and limited capacity for ongoing care.

### Overview of SOAR Training

Beginning in 2001, with the support of the Substance Abuse and Mental Health Services Administration (SAMHSA), Policy Research Associates, Inc. (PRA) developed SAMHSA’s *Stepping Stones to Recovery* manual and training curriculum, which is the basis for training in the SOAR model.

Since SOAR began, 27,226 people who were experiencing or at risk for homelessness have been approved for SSI/SSDI upon initial application through the SOAR approach. An additional 4021 persons, whose applications were denied initially, have been approved on reconsideration or appeal. Taken together, since 2006, the SOAR approach is responsible for assisting 31,247 persons to access Social Security disability benefits. The average approval rate on initial applications is 65%, with decisions received in an average of 94 days in 2014 and 81 days in 2015 (12).

A significant component of the SOAR approach is training direct service staff to increase their effectiveness in completing SSI/SSDI applications. Training is currently available in two forms: in-person and online. The in-person, 2-day training is delivered by local SOAR trainers who attended a SAMHSA-sponsored, PRA-provided, train-the-trainer (TTT) program between 2004 and 2013. Since 2004, PRA has trained 1004 trainers who conducted 1318 SOAR trainings.

Despite significant resources dedicated to the in-person training model, an external evaluation of SOAR found that only 13% of trainees who received the in-person training were actually assisting with applications post-training (13). This corresponds with other research documenting that in-person trainings result in lower student performance compared with online training (14). We know that some proportion of those trainees never assisted an individual experiencing homelessness with SSI/SSDI because they were intimidated by the idea of doing their first application. The SOAR Online Course addresses this particular barrier and opens up new opportunities to build system capacity to end homelessness in states and communities.

### Transition to the SOAR Online Course

The SOAR online training content and website were developed between 2010 and 2013. Pilot and beta testing of the training extended throughout 2014, and course content was revised based on feedback. Specific improvements included clarifying terms and fixing typos in course articles, re-ordering the presentation of information, and addressing technical difficulties. The course was released to the public in October 2014 after final review by SAMHSA and SSA staff and in conjunction with the start of a 5-year SAMHSA contract for the SOAR TA center (base year and 4 option years). While the contract year began from September 15, 2014, the authors evaluated activity from October 1, 2014 to September 30, 2015, with the sole exception of the trainee survey, explained below.

The SOAR Online Course consists of seven classes, each of which has a series of articles, videos, short quizzes, and a practice case. The course trains individuals in the core elements of completing SSI/SSDI applications using the SOAR model and includes a practice case. The practice case provides an opportunity for case managers to apply what they have learned by completing an SSI/SSDI application packet for a fictitious applicant. Video interviews, medical records, and progress notes provide the information needed to complete SSA forms and write a medical summary report (MSR). The packet is submitted to the SOAR TA center for review, and each application receives extensive written feedback. Upon approval, the participant receives a certificate of completion and 16 continuing education units (CEUs) from the National Association of Social Workers (NASW). We estimate that it takes about 16 h to complete the entire course with participants working at their own pace. However, we encourage students to complete the course within 30 days to retain the information learned and get connected sooner to local SOAR initiatives.

Studies show that students retain more information when they involve active learning, such as generative activities, whereby they must apply the information learned, self-assessment and reflection, and increased time to review and re-read course materials (14–17). The course and practice case allows trainees the flexibility to complete the training at their own pace and review sections or return to it whenever they need to brush up on a particular topic. While the addition of a practice case may result in fewer people completing the online training than those who attended in-person trainings, this active learning approach will better prepare trainees to assist with SSI/SSDI applications.

The online training brings standardization to the curriculum and ensures that all SOAR-trained providers have received the same basic training. Course coordinators review the online curriculum monthly and update content according to changes in SSA regulations. As a result, trainees receive the most up-to-date information on the disability process. By ensuring that the training is always up-to-date and providing timely, comprehensive feedback and technical assistance to trainees, the course is designed to attract and retain trainees based on guidance provided by Ballew et al. (18).

SSI/SSDI Outreach, Access and Recovery community leaders and state team leads (STLs) can follow-up with tailored 1-day trainings to provide information specific to their state’s SOAR process. Some leads also form facilitated online training cohorts, whereby they guide a group of participants through the course. This can include weekly check-in calls to assess trainee progress and understanding. A meta-analysis of online training programs found that student performance in online training settings was highest in courses that blended online and in-person training (14).

The online training is accessible wherever there is an Internet connection, thus expanding the impact of SOAR to rural and other communities that have not had previous access to trainings. Ballew et al. (18) found that online trainings increase access and flexibility for trainees but noted that lack of reliable Internet service or updated technology is still a barrier in fully implementing online training in all areas.

While the literature documents the need for increased interactivity, beyond a study evaluating training utilizing virtual three-dimensional environments (19), there are no evaluations analyzing the use of mock applications in an online setting to increase skills for social service providers. This evaluation intends to fill this gap and analyze the effectiveness of incorporating a realistic and multimodal practice case application with comprehensive feedback provided by experts, while also facilitating the completion of the online training through live guided cohorts.

## Evaluation Methods

Statistics used for the evaluation were gathered from October 1, 2014 through September 30, 2015 (base year). We analyzed the activity and outcomes from all course users who either completed the course or enrolled in the course during this time frame (*n* = 4477 users).

In the base year, 1140 individuals completed the course and submitted documents to the SOAR TA center for review; of these individuals, 1049 successfully passed the course. A majority, 645 trainees (56.6%), passed on the first submission and 404 trainees (35.4%) passed on a second, revised submission. Eleven individuals were unable to successfully complete the course, and packets for 80 individuals were either incomplete, under review, or had been reviewed with revisions requested and were still pending. A further 3337 individuals enrolled in the course during the base year but did not complete the training within the time period under evaluation.

For the practice case, participants are randomly assigned at equal rates a male or female case study. There was no significant difference in the pass rates between those who received the male or female case study. As a result, this evaluation did not explore differences in the case studies themselves that may have impacted course outcomes.

We used a multipronged approach to evaluate quantitative data on course usage and the translation of learning to practice, and qualitative data about user experience. To gather the course usage data, we utilized Google Analytics for website and course page use, YouTube Analytics to assess course video use, and mapped course completion rates from the SOAR database that tracks course progress and results. We sought to assess: (1) the number of people who looked at the course website compared to enrollment statistics, (2) the geographic reach of the course, (3) differences in completion rates for individuals who have a nearby SOAR Lead compared to areas without a SOAR Lead, (4) average length of time to completion from course enrollment to practice case submission, and (5) utilization of course materials.

To gather qualitative data about user experience, we delivered surveys *via* SurveyMonkey to gather information about the characteristics of who is completing the course and responses about course content. The survey was sent to the individuals who passed the course during the dates of the FY2015 contract year for the SAMHSA SOAR TA center, September 15, 2014–September 14, 2015. The survey link was sent to 863 people who had completed the review process for the practice case and successfully passed the course in that time frame. The evaluation survey was then sent to everyone who passed or failed ongoing from September 15, 2015 to track feedback for FY2016. The survey responses are anonymous, so we are unable to identify the people who received and potentially responded to the survey in the 2 weeks left out of the evaluation period (September 15–30, 2015) to add them in and the 2 weeks prior (September 15–30, 2014) to remove them. This partially explains the discrepancies in our course completion totals and survey totals. The balance can be explained by the fact that after participants submit their practice case applications for review (i.e., complete the course), they may be required to revise and resubmit forms. The time required for resubmission and additional review can cause their original submission and pass/fail dates to fall into two different fiscal years.

The survey asked respondents to provide detail about their background with eight multiple choice questions (agency type, role, funding source, current application assistance outcomes, and cohort participation). Respondents were asked to measure their agreement on a 4-point scale with 19 statements relating to the online course (content comprehension, course content, and the practice case component). Two additional questions asked respondents to rate their satisfaction on a 5-point scale regarding information, organization, and presentation of course materials. Finally, the survey included three open-ended questions about likes, dislikes, and suggested improvements to the course.

Further, we cross-referenced those who successfully completed the course during the evaluation period with SOAR Online Application Tracking (OAT) system outcomes to compare SSI/SSDI application outcomes between those who received the online training and those who took the in-person training. The SOAR OAT program is online, HIPAA-compliant, and accessible from any web-enabled device. It is free to access, user-friendly, and asks as few questions as possible to minimize data entry burden. While not all SOAR programs utilize the OAT system to track outcomes, data were sufficient to analyze trends between the training methods.

These evaluation methods were undertaken to address five research questions:

1. What types of caseworkers are utilizing the SOAR Online Course?
2. What benefits and barriers can we identify by using a practice case component in the online training?
3. Does facilitated training (i.e., cohorts and follow-up) increase participation in the training?
4. Does online training increase the quality and quantity of submitted SSI/SSDI applications compared to in-person training?
5. How can these results be expanded into other areas of online training for social services?

## Evaluation Findings

### Enrollment Statistics and Attrition during Base Year

The evaluation found high attrition rates in the SOAR Online Course, with only 25.43% of total enrollees completing the course during the base year. This is consistent with previous studies that found high attrition rates in free, non-facilitated online courses due to a number of people enrolling in the course as “window shoppers,” without intentions of completing the training (20). In addition, lack of organizational support during the training and lack of relevance to a learner’s job have been found to predict increased course dropout rates (21). This current study did not evaluate the reasons behind the high attrition rate for the SOAR Online Course enrollees, and as Stone (20) notes, future research is needed in this area.

### Course Usage Mapping and Correlation with SOAR Leaders

To evaluate course reach and to examine possible correlations between successful course completions and the presence of a local SOAR leader, we created a map with three sets of data: number of enrolled online course users by state, successful course completions by zip code, and the location of a local SOAR leader by city and state. The information about course enrollees and completions was pulled from an internal database for the SOAR Online Course. Location information for SOAR leaders was gathered from an internal database of individuals who attended a 3-day SOAR Leadership Academy, sponsored by SAMHSA, in the 2-year period before the end of the base year. This time frame allowed us to map any potential impact SOAR leaders have on usage of the SOAR Online Course.

**Figure 1:**
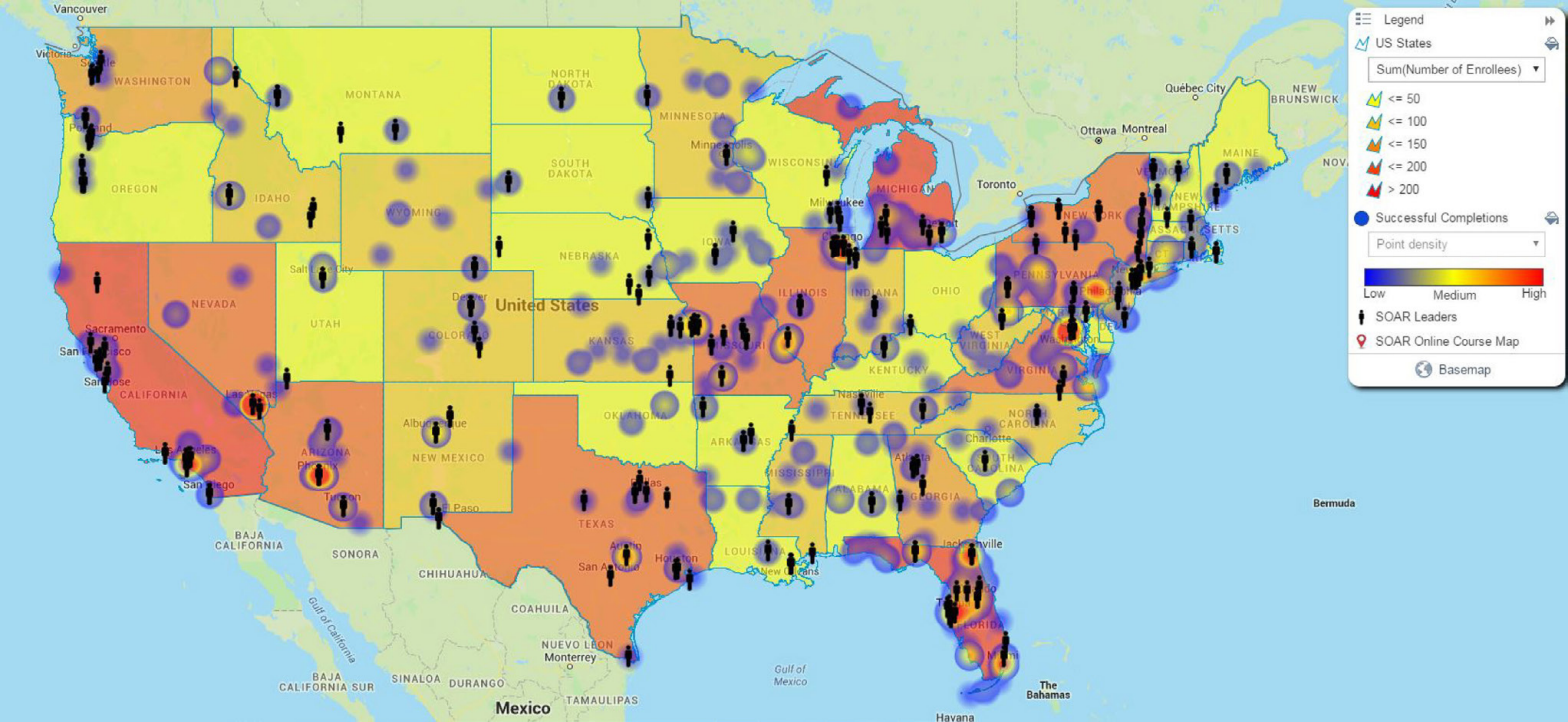
**SOAR Online Course enrollments, completions, and locations of SOAR Leads**.

Figure 1 shows the distribution of successful completions by state through shading gradients, the number of successful course completions by zip code using blue to red heat mapping, and the locations of SOAR leaders by city using icons. Alaska and Puerto Rico are not pictured, but each had three successful course completions in the base year and do not have any SOAR leaders. Hawaii was excluded from Figure 1, as it also had an insignificant amount of SOAR Online Course activity during the evaluation period.

Through mapping the course usage, we tested our hypothesis that the SOAR Online Course would expand the training to new areas around the country. All states had enrollees in the course and trainees from 49 states, the District of Columbia, and Puerto Rico successfully completed the course during the base year. Ohio did not have any providers complete the SOAR Online Course in this timeframe.

The significant finding in this map is that successful course completions are clustered in the areas that have local SOAR leaders, particularly in the areas with high concentrations of completions. Through the Leadership Academy, SOAR leaders are trained on how to facilitate groups through the course and support trainees, and this may be a key contributing factor to this finding. Evaluating the causal link between these factors was outside the scope of the current study, though future research into this area would be beneficial.

### Days to Successful Completion

To determine the length of time to complete the course, we calculated the days from course enrollment to the date the course packet was submitted. We matched the days to completion with the role the trainee most identified with during their course registration: agency director, case manager, local SOAR lead, peer specialist, SOAR state team lead, or personal use. The latter category indicates that the trainee was taking the course to help himself or herself or a friend/family member.

We also evaluated who was successfully completing the SOAR Online Course, by role, during the base year. The majority of successful completions (52%) were by those who self-identified as a case manager. State, local, and agency leads represent a minority of course completions and may be reflective of supervisors taking the program before introducing it to their staff. Of note, 360 trainees (34.3%) left the role blank.

As Table 1 illustrates, SOAR Online Course trainees take approximately 44 days to complete the training, and case managers represent over half of successful completions.

**Table 1:** **Average days to completion of the SOAR Online Course by role**.

Role	Number of completions	Percentage of total successful completions	Average days to completion
Agency director	61	6	43.1
Case manager	544	52	45.7
Local SOAR lead	29	3	44.2
Peer specialist	36	3	35.6
SOAR state team lead	5	<1	78
Personal use	14	1	55.8
Other (role left blank)	360	34	41.7
Grand total	1049	100	44.1

### Utilization of Course Materials

Each of the two practice cases in the SOAR Online Course has three video interviews with the mock applicant. Watching these videos in full is a key to successful completion of the training. To evaluate the utilization of the course videos, we used YouTube Analytics gathered from the base year to determine the average percentage that each course video was viewed. Table 2 presents these findings.

**Table 2:** **Data and analysis of the SOAR Online Course YouTube videos**.

Video segment	Full duration of video	Average percentage of video viewed	Number of viewers
Male video 1	5:15	102.47	2276
Male video 2	3:50	93.71	1434
Male video 3	4:50	92.2	1087
Female video 1	8:29	88.34	2127
Female video 2	6:22	89.16	1433
Female video 3	9:44	85.91	1198

		**Video length**	**Percent viewed**
**Statistics**			
Mean		0:06:39	89.8640
SEM		0:01:05	1.39106
Median		0:06:22	89.1600
Mode		0:03:50^a^	85.91^a^
SD		0:02:27	3.11050
Variance		21,764.000	9.675
Range		0:05:54	7.80
Minimum		0:03:50	85.91
Maximum		0:09:44	93.71
Percentiles	25	0:04:20	87.1250
	50	0:06:22	89.1600
	75	0:09:06	92.9550
**Pearson product-moment correlation**			
Video length	Pearson correlation	1	−0.976^b^
	Sig. (2-tailed)		0.004
	*N*	5	5
Percent viewed	Pearson correlation	−0.976^b^	1
	Sig. (2-tailed)	0.004	
	*N*	5	5

*^a^Multiple modes exist. The smallest value is shown*.

*^b^Correlation is significant at the 0.01 level (2-tailed)*.

*Coefficient of determination (*r*^2^) = 0.953*.

A Pearson product-moment correlation was run to determine the relationship between the length of the practice case videos and the percentage of the video watched. The data showed no violation of normality, linearity, or homoscedasticity, after one outlier (male video 1) was removed prior to calculations. The male actor in video #1 spoke quickly and with a strong accent, which may have led to difficulties in understanding him, requiring repeated viewing. This may account for the high percentage of views for the male video #1 compared to the others.

There was a strong, negative correlation between length of video and percentage viewed, which was statistically significant (*r* = −0.976, *n* = 5, *p* < 0.005), resulting in a finding that an increase in video length is correlated with a decrease in percentage of the video viewed.

### Qualitative Findings about User Experience

In October 2015, the SOAR TA center surveyed individuals who successfully completed the course during the base year. Surveys were delivered *via* SurveyMonkey to 863 individuals to gather background information and characteristics of participants completing the course and quantitative and qualitative responses to the course content and self-assessed comprehension. One hundred and thirty-six people responded to the survey (15.76% response rate), and the results are below in Table 3.

**Table 3:** **Respondent characteristics from the survey of base year trainees**.

	*n*	%
**Agency**
Community mental health agency	45	33
Homeless service agency	39	29
Veteran services agency	4	3
State agency	9	7
Other	39	29
**Position**
Case manager	51	38
Outreach worker	10	7
Shelter worker	1	1
Benefits specialist	13	10
Program coordinator/supervisor	23	17
Other	38	28
**Other characteristics**
Participated in the SOAR Online Course as part of a facilitated local training group/cohort	46	35
Planning to attend a local SOAR Fundamentals refresher training after the online course	48	37
Assisted adults with SSI/SSDI applications in the past year	50	37

Table 4 displays the results of the SOAR Online Course evaluation portion of the survey. The majority, 96% of respondents, agreed or strongly agreed with the statement: “Overall, I feel this training will help me do a better job assisting individuals with SSI/SSDI applications.” Similarly, 96% of respondents were satisfied or very satisfied with the information provided in the SOAR Online Course. Note that for this portion of the survey, *n* = 122, as 14 participants did not respond to these questions.

**Table 4:** **SOAR Online Course evaluation results**.

SOAR Online Course evaluation questions	% agree/strongly agree (*N*)
**Content comprehension**	
1. I have a better understanding of the differences between SSI and SSDI, including the health insurance offered and eligibility requirements	92 (113)
2. I am better able to identify the non-medical criteria for SSI/SSDI eligibility	91 (112)
3. I have a better understanding of the disability determination process and how to develop medical evidence to support a disability claim	97 (119)
4. I have a clearer understanding of the role of functional information in the determination of disability	97 (118)
5. I feel more equipped to thoroughly interview an SSI/SSDI client and assess his/her functioning	94 (114)
6. I will be able to write a comprehensive medical summary to be submitted for disability determination	96 (117)
7. Overall, I feel this training will help me do a better job assisting individuals with SSI/SSDI applications	96 (117)
**Course content**	
8. The course was comprehensive and the information was up-to-date	93 (113)
9. The course was interesting and held my attention	84 (103)
10. The course presented the information clearly	85 (103)
11. The course was organized in a way that was conducive to learning	87 (106)
12. The balance of information among written materials, videos, and the practice case was effective	90 (110)
13. The tools and worksheets reference in the course will be useful for my work in the field	91 (111)
**Practice case component**	
14. The practice case helped me to better understand the material being covered in the course	94 (115)
15. Completing the practice case helped me explore how I will use the information in my own work	91 (111)
16. The materials provided for my “applicant” were sufficient to complete the required SSA forms and the SOAR MSR	90 (110)
17. The feedback provided was helpful in my learning and understanding	94 (114)
18. The feedback was provided in a timely manner	93 (114)
19. I feel more confident about assisting with an actual SSI/SSDI application having completed the practice case	94 (114)

A majority of respondents (63%) indicated that prior to completing the SOAR Online Course they had not assisted with any SSI/SSDI applications over the past year, while 23% indicated that they assisted with less than a single application per month. Over half of respondents who assisted with SSI/SSDI applications had less than a 25% approval rate prior to taking the training, with almost a quarter of respondents having a 0% approval rate. Notably, after completing the SOAR Online Course 94% of respondents agreed with the statement, “I feel more confident about assisting with an actual SSI/SSDI application having completed the practice case.” As discussed below, a significant percentage of the evaluation period trainees registered for the SOAR OAT system and began documenting positive outcomes after completing the training.

Additionally, survey respondents were asked about the two things they liked the most and the least about the SOAR Online Course. The “most liked” features of the course question generated 200 comments from 102 respondents. The top two responses were the practice case component (23%, 45 comments) and the comprehensive information in the course (16%, 32 comments). Other well-liked features included the videos throughout the course and the feedback and support from the SOAR TA center.

The question asking what participants disliked the most about the course generated 146 comments from 90 respondents. Twenty comments were categorized as “not applicable” as they contained positive comments, “n/a,” or “none.” The top two responses after this indicated that the course is too long (10%, 14 comments) and that respondents did not like the online setting of the course (9%, 13 comments). Other disliked features of the course included confusion in how to get started with the course, writing a MSR, and too much commitment required to complete the training.

### Quantitative Results of SOAR Outcomes from Trainees

During the base year, 151 trainees who successfully completed the course registered for the OAT system. Of these trainees, 64 entered outcomes for SSI/SSDI applications into the system. This 42% data entry rate is much higher than from those who received the in-person training during the evaluation period. By contrast, of the 210 individuals who received the in-person training and registered for the OAT system during the evaluation period, only 65 trainees (31%) entered data into OAT.

Providers who took the online course documented outcomes on 405 initial applications for SSI/SSDI, with an average approval rate of 71%, 6% points higher than the national SOAR average approval rate of 65%. Providers reported receiving the decisions in 91 days, compared to national averages of 94 and 81 days in 2014 and 2015, respectively. SOAR Online Course trainees also assisted with 116 appeals, which include reconsiderations and ALJ hearings. Sixty-one percent of these appeals were approved, which compares to a national average for SOAR-assisted appeals of 52%.

Interestingly, the approval rates for both SOAR-assisted initial applications (71%) and appeals (61%) were the same for the group who received in-person training and registered for OAT during the base year. This group completed 495 initial claims and 146 appeals. The key difference was that this group averaged 109 days to decision for initial claims. However, it is unclear what role training modality had in this outcome, as differences could be due to varying decision times in the provider’s regions.

## Discussion and Implications

The SOAR Online Course was developed to expand the reach of SOAR training, ensure standardized curriculum, and increase the capacity for public health workers to assist with SSI/SSDI applications. This evaluation aimed to address five key research questions.

### What Types of Caseworkers Are Utilizing the SOAR Online Course?

We found that the majority of trainees who successfully completed the course self-identified as case managers, while a minority identified as state, local, or agency leads. Combining the case manager role with peer specialists, direct service workers account for 55% of all successful completions. This figure corresponds to the general structure of social service roles, with more direct service providers than oversight roles. It also corresponds well with the marketing of the course. As the aim of the course is to provide training in direct SSI/SSDI application assistance, we gear marketing toward direct service providers. We provide additional materials that are aimed toward supervisors to gain buy-in for the program, but this focus in on review of the course rather than full completion of the practice case.

We found that 34% of users left the role item blank during registration. In order to better understand the intended use of enrolled participants, PRA modified the enrollment for the online curriculum to include a question indicating the user’s intention with the curriculum for future evaluation. Enrollees entering the course after the base year will indicate whether they are going to take Class 1 as an overview, look at particular articles as a refresher training, fully complete the course, or just browse for additional information. This will provide greater detail about why some individuals are enrolling but not completing the course. Other online training programs would benefit from including these types of registration questions during program launch, to more fully understand trainee intent.

### What Benefits and Barriers Can We Identify by Using a Practice Case Component in the Online Training?

The practice case component was a unique addition to the course during the transition to online training from in-person curriculum. This evaluation found that this component introduced a number of barriers and benefits for the training.

The practice case component does not allow the training participant to passively participate as they might in an in-person didactic presentation. It requires additional time, concentration, and investment to complete the activity. This barrier causes participants to self-select out of full completion of the online training because they do not have the time, the need, or do not see the value in successful completion. The high attrition rate with the SOAR Online Course is in line with previous studies; however, an additional barrier we found leading to high dropout rates, is the inability of case workers to bill for their time while completing the course.

The survey of trainees supports these findings, with many of the negative comments about the course relating to the length, time commitment, and the need to write a four to six page report as part of the practice case. Other online training programs should take into account the need for organizational support and flexibility in billing practices when integrating a practice case component into the training. This support is critical in retaining those trainees who could benefit from the course and reduce disincentives for taking additional training in the social service sector.

While there are some barriers to including the practice case component in the online course, this evaluation revealed benefits to integrating these generative learning activities. Including a realistic practice case that tests comprehension and application of information provided in the training helps training administrators know whether participants have increased their knowledge and benefited from the course. Integrating individual review of practice case materials and extensive feedback and technical assistance from the SOAR TA center ensures that trainees must fully understand the SSI/SSDI application process before successfully completing the course. For those trainees who do not pass the course on the first attempt and need additional revisions to their packets, reviewers provide comprehensive guidance and explanations. This additional support from national experts was not previously found in the in-person training model and provides an additional benefit to trainees.

Results from the qualitative surveys and feedback from the base year survey indicate strong positive views of the utility of the SOAR Online Course. The majority of respondents in the base year survey had not assisted with SSI/SSDI applications prior to the training but indicated an increase in confidence to do so after completion of the course. As shown below, many trainees from the evaluation translated this training into practice with SSI/SSDI applications. This finding presents an opportunity to overcome the barrier that in-person trainees faced, previously leading to a low number of applications post-training.

### Does Facilitated Training (i.e., Cohorts and Follow-up) Increase Participation in the Training?

When analyzing course completions by location, we found that successful course completions were concentrated in Florida, Virginia, California, and Nevada. The concentration in California and Florida may be explained by the high number of enrolled users in both states (465 and 638, respectively). Virginia and Nevada have relatively low numbers of enrolled users (179 and 153, respectively); however, the local and state leads in those areas facilitated training groups that helped individuals through the course, leading to a higher completion rate relative to the number of enrolled users.

The course usage mapping showed a high correlation between those areas that have local SOAR leaders and successful online course completion. While evaluation of the causal effect of local facilitators on completions was outside the scope of this study, the results indicate a positive connection between the two factors. SOAR leaders are taught to facilitate groups of trainees through the course, provide technical assistance on the training and SOAR process, and use blended training methods of the online course with in-person follow-up. These factors, along with the fact that SOAR leaders play a key role in the marketing of the course locally, may account for results found during the mapping process.

This indicates that utilizing the training cohort model developed by PRA and having local SOAR leaders to facilitate training can be effective in increasing course completion rates. Additionally, the data mapping indicates that the course has a wide reach across the United States and its territories, indicating that information about the training is expanding to new areas; however, future work will focus on increasing course completions and building capacity in rural areas.

### Does Online Training Increase the Quality and Quantity of Submitted SSI/SSDI Applications Compared to In-Person Training?

Our evaluation results indicated that a higher percentage of trainees entered data into the OAT system if they were trained through the SOAR Online Course, as opposed to the in-person training model (42 versus 31%). While the sample sizes for both groups were relatively small (151 and 210, respectively), this indicates a positive trend that should be explored in future evaluations. One possible reason for the higher data entry rate may be the strong emphasis in the SOAR Online Course on tracking outcomes and the OAT system. This material is not part of the in-person training curriculum and dissemination of information about OAT is at the discretion of individual trainers.

We found that the approval rates for both initial applications and appeals were the same between the two training modalities. The in-person training group assisted with more applications than the online training group during this period; however, the small sample size in this aspect of the evaluation limits our ability to extrapolate these findings and assess any significant changes in quantity or quality of applications. One limitation of these data is that we were only able to evaluate outcome results that were entered into the OAT system. We do not have internal access to individual outcomes that are entered into state-level databases or homeless management information systems (HMIS).

### How Can These Results Be Expanded into Other Areas of Online Training for Social Services?

Other factors to be considered when designing an online training program is the length of time it takes for trainees to complete the course, particularly compared to in-person trainings. We found that those who successfully completed the course did so in an average of 44 days. An exception to this is the state lead role, which has an average of 78 days to completion of the course. This may be attributed to the competing demands of state leads, as they are often leading other statewide initiatives, such as SAMHSA’s Projects for Assistance in Transition from Homelessness (PATH).

Results from this evaluation indicate that trainees need more time to complete the course than advertised on the course website. The SOAR Online Course marketing materials estimate that trainees can complete the course in approximately 16 h over 30 days; however, this was insufficient for many users. The discrepancy between advertised length and actual length may attribute to some of these negative views about course length. While removing the practice case element may shorten the training time, this evaluation finds that the benefits of including this active learning component outweigh the barriers for those who can commit the time. However, other social service trainings may not require as extensive a practice case as the one utilized by the SOAR Online Course, due to the intensive nature of SSI/SSDI applications. As a result, other trainings could reduce the amount of training time required to meet the programmatic needs.

In analyzing the course video utilization, we found that the length of video had a strong, negative correlation to percentage of the video that was viewed. As a result, the longer videos in our training curriculum are less likely to be viewed in their entirety. Trainees who do not watch videos in full miss out on key elements of the course. The course video that received the highest percentage of view time, excluding the outlier male video #1, was 3 min and 50 s in length. This indicates that when creating course, content videos should be no longer than 4 min each, with shorter videos receiving more complete views.

Some study limitations merit comment. The base year surveys were only sent to those who successfully passed the course. Future research designs including individuals who enrolled in the course, but did not complete the training, would provide a better understanding of barriers to course completion. Additionally, the base year survey was delivered in October 2015 and may have had a response bias toward those who most recently completed the training. We addressed this potential response bias by issuing course surveys to trainees as soon as they successfully complete the training beginning in December 2015.

## Conclusion

As shown above, our evaluation demonstrates that an online training course for social service providers can be effective in increasing capacity for assisting vulnerable individuals. The SOAR Online Course expands training opportunities for individuals to learn how to effectively assist with SSI/SSDI applications for individuals experiencing or at risk for homelessness.

This evaluation revealed that key success factors for online training models include the integration of a practice case component (or other generative learning activity), support from local facilitators, and feedback and technical assistance for trainees. Further evaluation is needed regarding reasons behind high attrition rates in the course and causal links between facilitation and successful course completion; however, results indicate the potential for other public health providers to utilize online training models to increase knowledge and capacity.

## Author Contributions

Both authors made substantial contribution to the design and concept of the evaluation, and drafted the manuscript under review. KL contributed toward background research, narrative information about the course, analysis from the pilot testing phase of the evaluation, and discussion of the findings. JE contributed to the analysis and interpretation of base year study data, and overall implications and discussion of the findings. Both authors give final approval for the draft to be published and agree to be accountable for all aspects of the work in ensuring that questions related to the accuracy or integrity of any part of the work are appropriately investigated and resolved.

## Conflict of Interest Statement

This research was conducted in the absence of any commercial or financial relationships that could be construed as a potential conflict of interest.

## Abbreviations

OAT, Online Application Tracking; PRA, Policy Research Associates, Inc.; SAMHSA, Substance Abuse and Mental Health Services Administration; SOAR, SSI/SSDI Outreach, Access and Recovery, SSA, Social Security Administration; SSDI, Social Security Disability Insurance; SSI, Supplemental Security Income; STL, state team lead.
